# The Orexin-A/OX1R System Induces Cell Death in Pancreatic Cancer Cells Resistant to Gemcitabine and Nab-Paclitaxel Treatment

**DOI:** 10.3389/fonc.2022.904327

**Published:** 2022-06-07

**Authors:** Thierry Voisin, Pascal Nicole, Valérie Gratio, Anaïs Chassac, Dounia Mansour, Vinciane Rebours, Anne Couvelard, Alain Couvineau

**Affiliations:** ^1^ INSERM UMR1149/Inflammation Research Center (CRI), Université Paris Cité, Team “From inflammation to cancer in digestive diseases” labeled by “la Ligue Nationale Contre le Cancer”, DHU UNITY, Paris, France; ^2^ Department of Pathology, Bichat Hospital, Université Paris Cité, Paris, France; ^3^ Department of Pancreatology, Beaujon Hospital, Université Paris Cité, Clichy, France

**Keywords:** GPCR, pancreatic cancer, orexins, chemoresistance, apoptosis

## Abstract

Pancreatic ductal adenocarcinoma (PDAC) represents the fourth cause of cancer-associated death in the West. This type of cancer has a very poor prognosis notably due to the development of chemoresistance when treatments including gemcitabine and Abraxane (Nab-paclitaxel) were prescribed. The identification of new treatment circumventing this chemoresistance represents a key challenge. Previous studies demonstrated that the activation of orexin receptor type 1 (OX1R), which was ectopically expressed in PDAC, by its natural ligand named orexin-A (OxA), led to anti-tumoral effect resulting in the activation of mitochondrial pro-apoptotic mechanism. Here, we demonstrated that OxA inhibited the pancreatic cancer cell (AsPC-1) growth and inhibited the tumor volume in preclinical models as effectively as gemcitabine and Nab-paclitaxel. Moreover, the combination therapy including OxA plus gemcitabine or OxA plus Nab-paclitaxel was additive on the inhibition of cancer cell growth and tumor development. More importantly, the treatment by OxA of chemoresistant tumors to gemcitabine or Nab-paclitaxel obtained by successive xenografts in mice revealed that OxA was able to induce a strong inhibition of tumor development, whereas no OxA resistance was identified in tumors. The OX1R/OxA system might be an innovative and powerful alternative treatment of chemoresistant PDAC.

## Introduction

Pancreatic cancers widely represented (>90%) by pancreatic ductal adenocarcinoma (PDAC) are the most lethal human digestive cancer with a very poor 5-year-survival rate, which is <10% (1). To make matters worse, the statistical projection predicts that this cancer could become the second leading cause of cancer mortality in the next decade (2). Moreover, this sad description was mainly associated with the difficulty of early diagnosis of the disease, the risk of tumor recurrence after surgery, and the development of resistance to various therapies (3, 4). Currently, the major therapy for unresectable advanced PDAC was regrouped into various combinatorial protocols used in first/second line of treatment and dependent on the countries that mostly based on Nab-paclitaxel/gemcitabine or FOLFIRINOX (folinic acid, 5-FU, irinotecan, and oxaliplatin) (5). In the last decade, gemcitabine (2′-desoxy-2′,2′-difluorocytidine), a deoxycytidine analog, which is a DNA synthesis inhibitor able to block cell cycle and cell proliferation, was mainly used in first-line treatment of PDAC (6). After intracellular phosphorylation, gemcitabine was transported and incorporated into DNA by various transporters and enzymes to induce their deleterious effects in cells by competition with deoxycytidine triphosphate (6). However, PDAC patients rapidly develop chemoresistance to gemcitabine, which strongly reduced the treatment effectiveness compared to other chemotherapeutic molecules (6). While the mechanism responsible for gemcitabine resistance remains broadly unclear, many studies have demonstrated that gemcitabine metabolism involved nucleoside transporters and enzymes (3, 7). Moreover, the role of epithelial–mesenchymal transition (EMT) that represents a phenotypic change in cancer cells leading to higher cell aggressiveness also play a role in gemcitabine-resistant mechanisms (3). Other mechanisms involving factors secreted in the tumor microenvironment by pancreatic stellate cells, fibroblasts, nerve cells, endothelial cells, exosomes, and inflammatory cells were reported (3, 6). Moreover, the inactivation of pro-apoptotic process, the alteration of the cell cycle, the cell stress modifications, the inhibition of DNA repair systems, the inactivation of important coding genes by genetic and/or epigenetic modifications, the amplification/deletion of miRNA genes associated or not to a dysregulation of miRNA transcription, and the overexpression of certain long non-coding RNAs (lncRNAs) were also identified as factors inducing drug resistance (8, 9). The combination of gemcitabine to Nab-paclitaxel (Trade name: Abraxane), which consisted of albumin nanoparticles (nab) associated to paclitaxel, in neoadjuvant treatment before surgery and in advanced metastatic PDAC has demonstrated a significant survival gain compared to gemcitabine monotherapy (10, 11). Paclitaxel belonging to the taxane family was an anti-mitotic drug blocking the microtubule depolarization leading to the inhibition of mitosis (12). Although lesser studied, the development of Nab-paclitaxel resistance seems to be related to drug metabolism involving oxidative reactions (13).

In this context, identification of new targets with antitumoral action on drug-resistant cancer cells represents an essential challenge. Since several years, our group demonstrated that orexin receptor type 1 (OX1R) but not orexin receptor type 2 (OX2R) was expressed in digestive cancer cells including pancreatic, colon, and liver cancers (14) where its activation by orexins induced a mitochondrial apoptosis leading to anti-tumoral impact of orexin/OX1R system (15). Orexins specifically activated two receptor subtypes belonging to the G-protein-coupled receptor (GPCR) family (16). Orexins, encompassing two isoforms named orexin-A (OxA) and orexin-B (OxB), are hypothalamic neuropeptides produced by the same precursor, the prepro-orexin (16). The main biological function of orexins was to control the wakefulness (17), and a dysregulation of this function led to human narcolepsy type I pathology (18). In 2018, it has been reported that OX1R was expressed in 96% of PDAC and also in pancreatic precancerous lesions (PanIN), whereas this receptor was not expressed in healthy pancreas exocrine tissue (19). Activation of OX1R by OxA in pancreatic cancer cells induced mitochondrial apoptosis (19). The orexin-inducted apoptosis was mediated by the phosphorylation of two immunoreceptor tyrosine-based inhibitory motifs (ITIMs) present in the OX1R sequence, the recruitment of the tyrosine-protein phosphatase non-receptor type 11 (SHP2), the activation of p38 mitogen-stress protein kinase leading to the translocation of proapoptotic Bax protein in mitochondria, releasing of cytochrome c, and activation of caspase 3 and 7 (20). In preclinical mouse models subcutaneously xenografted with the pancreatic cancer cell line, AsPC-1 or with isolated cells from PDAC patient named patient-derived xenografts (PDX), intraperitoneal injection of OxA induced a strong reduction in tumor volume (19). Based on these observations, the aim of the present study was to compare the anti-tumoral action on pancreatic cancer cell line, AsPC-1, of OxA to the action of gemcitabine and Nab-paclitaxel. Moreover, OxA is able to induce a strong inhibition of tumor growth in preclinical mouse models xenografted with pancreatic cancer cells made resistant to gemcitabine or Nab-paclitaxel treatment. These data demonstrate that the OxA/OX1R system represent a therapeutically innovative target in PDAC treatment notably in drug-resistant pancreatic cancers.

## Materials and Methods

### Cell Culture

PDAC cell line, AsPC-1 (ATCC, CRL-1682), was cultured at 37°C in 5% CO_2_/95% air in Roswell Park Memorial Institute (RPMI 1640) medium supplemented with 10% (v/v) fetal calf serum, 100 µg/ml streptomycin, 100 U/ml penicillin, and 1% (v/v) ZellShield (Minerva Biolabs, Berlin, Germany) to prevent mycoplasma contamination. AsPC-1 cells were incubated in the presence or in the absence of 0.1 µM orexin-A (OxA) (GL Biochemicals, Shangaï, China), 0.01 µM gemcitabine, 0.1 µM Nab-paclitaxel, 0.01 µM gemcitabine + 0.1 µM OxA, or 0.1 µM Nab-paclitaxel + 0.1 µM OxA. These doses of compounds corresponded to the dose that induced half of the effect on the cell growth inhibition. After 48 h of treatment, total cells were harvested and counted to determine the cell viability, as previously reported (19). To quantify apoptotic cells, the Guava Nexin Kit (Guava Technologies, Luminex, Austin, TX, USA) was used following the manufacturer’s procedure. The dose–response curves of OxA, gemcitabine, Nab-paclitaxel, and their combination action were analyzed with SynergyFinder (v2.0) stand-alone-web application (21 and https://synergyfinder.fimm.fi/). In sequential treatment, AsPC-1 cells were incubated in the presence of 0.1 µM orexin-A or 0.01 µM gemcitabine or 0.1 µM Nab-paclitaxel for 48 h. After pretreatment, compounds were substituted for another 48 h by 0.01 µM gemcitabine or 0.1 µM Nab-paclitaxel for cells pretreated by OxA and 0.1 µM OxA for cells pretreated by 0.01 µM gemcitabine or 0.1 µM Nab-paclitaxel, respectively. Control corresponded to the absence of treatment for 96 h.

### Immunohistochemical Procedure

Immunohistochemistry was performed on formalin-fixed paraffin-embedded 3-µm slices of spheroids or tumor tissue from xenografts with an automated immunohistochemical stainer according to the manufacturer’s guidelines (automate BOND, Leica Microsystems). Slides were immunolabeled with antibodies against OX1R (Life Technology, PA5-33837, polyclonal rabbit, 1/250) or activated caspase-3 (Abgent, E87-77, polyclonal rabbit, 1/100).

### Tumorigenicity Assays in Nude Mice Xenografts

A total of 2.10^6^ AsPC-1 cells or 2.10^6^ cancer cells isolated from pancreatic tumor patient were subcutaneously injected into the two flanks of five nude mice (to limit mouse number according to 3Rs rules) per arm corresponding to the development of 10 tumors per arm, as previously reported (19). At day 1 after cell injection, 100 µg OxA or 120 µg gemcitabine or 230 µg Nab-paclitaxel were intraperitoneally administered in 100 µl/injection three times per week until the endpoints defined in the protocol were reached. Control mice were intraperitoneally injected with phosphate-buffered saline (PBS). Tumor development was measured using a caliper according to a previous report (15). Briefly, the long and short axes (x,y) of tumors were determined three times per week, and tumor volumes were calculated according to the formula: V=(x.y2.π6) . After necropsy, subcutaneous tumors were resected, weighted, and histologically analyzed. All experiments were performed in accordance with European ethical laws and in particular respecting the 3Rs rule according Apafis No. 17199-201810221522166v4. All along tumoral developments, the general condition and pain of animals were monitored, and endpoints were scored. At the end of the experiments, all animals were euthanized by cervical dislocation. The use of human materials was approved by the Institutional Review Board (CEERB GHU Paris Nord Nos. IRB12-059 and 12-033).

### Establishment of Xenografted Resistant Tumors for Gemcitabine or Nab-Paclitaxel or OxA

To establish resistances to different compounds including PBS, gemcitabine, Nab-paclitaxel, or OxA, two successive runs of xenograft/treatment were planned. The first run (run01) consisted of subcutaneously inoculating AsPC-1 cells into nude mice followed by three intraperitoneal injections/week of PBS, 120 µg gemcitabine, 230 µg Nab-paclitaxel, or 100 µg OxA during 8 weeks before reaching the endpoints. Then, mice were euthanized, and tumors were resected. Tumors were sliced using scalpels and then incubated in dissociation medium containing culture medium added with collagenase IV (400 U/ml). The second run (run02) consisted of subcutaneously injecting dissociated cells corresponding to PBS-, gemcitabine-, Nab-paclitaxel-, or OxA-treated cells from run01 into a new batch of nude mice. Mice from run02 were again treated by three intraperitoneal 100-µl injections/week of PBS, 120 µg gemcitabine, 230 µg Nab-paclitaxel, or 100 µg OxA until endpoints defined in the protocol were reached. Tumor volumes were estimated as described above. At the end of the experiment, mice were euthanized and necropsied. Tumors were resected and weighted. Resected tumors were either formalin fixed or cell dissociated for RNA-seq and spheroid analyses.

### Spheroids Analysis

Spheroid formation was obtained from AsPC-1 cells resistant to gemcitabine or Nab-paclitaxel or OxA isolated from xenografted tumors obtained in run02 experiments (see above). Briefly, xenografted tumors corresponding to control, gemcitabine treated, and Nab-paclitaxel treated were collected and dissociated, and tumoral cells were grown in 96-well plate (Corning, NY, USA) containing Dulbecco’s modified Eagle’s medium (DMEM) supplemented with 4.5 g/L of glucose, 10% (v/v) fetal calf serum, 100 µg/ml streptomycin, and 100 U/ml of 3,000 cells/well under agitation at 200 rpm, and were treated or not with 1 µM OxA, 0.01 µM gemcitabine, 0.1 µM Nab-paclitaxel, 1 µM OxA + 0.01µM gemcitabine, or 1 µM OxA + 0.1 µM Nab-paclitaxel up to 10–15 days. The volume of spheroids was determined using the plugin SpheroidSizer (21) under MathLab environment. Cell viability of spheroids was analyzed with ReadyProbes cell viability Imaging Kit (ThermoFisher Scientific, Illkirch, France) in which NucBlue live reagent stained alive cell nuclei and NucGreen death reagent stained only death cell nuclei.

### RNA-seq Analysis

Transcriptomic analysis was performed on AsPC-1/gem^R^ or ASPC-1/pacli^R^ cells isolated from xenografted tumors acquired in run02 experiment (see above). After dissociation, tumor cells were cultivated in RPMI medium supplemented with 10% (v/v) fetal calf serum, 100 µg/ml streptomycin, 100 U/ml penicillin, and 1% (v/v) ZellShield in the presence of PBS (control), or 1 µM OxA for 24 h. Total RNA was extracted using Trizol reagent (Fisher Scientific, Illkirch, France) and then treated with DNAse I RNAse free (Fisher Scientific, Illkirch, France) to eliminate genomic DNA. The integrity of the total RNA was verified by agarose gel electrophoresis and quantified using NanoDrop One (Thermo Scientific, Les Ullis, France). RNA-seq was performed on genomic platform [Genomic Paris Centre (IBENS), Paris, France], and sequencing data were managed with the modular and scalable workflow engine, Eoulsan 2.5, which used DESeq 2 for normalization and differential analysis (IBENS, Paris, France). DESeq 2 files were analyzed with iDEP.94 (22), and the biological insight of mRNA significantly impacted by OxA was analyzed with WEB-based GEne SeT AnaLysis Toolkit (WebGeSTAT) packages using Kyoto Encyclopedia of Genes and Genomes (KEGG) database (https://www.genome.jp/kegg/). Heatmaps were generated with Morpheus (https://software.broadinstitute.org/morpheus).

### Immunoblotting Assay

Semiconfluent AsPC-1, AsPC-1 gem^R^, or AsPC-1 Nab-pacli^R^ cells were washed twice with phosphate-buffered saline and treated with 1 µM OxA in medium or with medium only at 37°C for 24 h. Cells were collected and lysed with RIPA buffer [10 mM Tris–HCl (pH 7.5), 150 mM NaCl, 1 mM EDTA, 1 mM EGTA, 2 mM Na_3_VO_4_, 0.1% sodium dodecyl sulfate (SDS), 1% Nonidet P-40, 1% sodium deoxycholate, 0.36 µg/ml phenylmethylsulfonyl fluoride, 0.01% soybean trypsin inhibitor, 0.01% leupeptin, and 0.01% aprotinin]. The mixture was gently agitated for 30 min at 4°C and centrifuged at 15,000×*g* for 15 min at 4°C, followed by collection of the supernatant. Solubilized proteins (100 µg) in Laemmli buffer (incubated for 5 min at 98°C) were loaded onto a 10% SDS-polyacrylamide gel, transferred to a nitrocellulose membrane, and immunoblotted with antibodies described below. Anti-SHP-2/SHPTP-2 (1:1,000) antibody was from Millipore (Guyancourt, France); anti-FoxM1 (D12D5) (1:1,000), anti-cleaved PARP (Asp214) (1:1,000), anti-PARP (46D11) (1:1,000), anti-elF2a (Ser51) (1:1,000 dilution), anti-ATF-3 (D2Y5W) (1:1,000 dilution), anti-ATF-4 (D4D8) (1:1,000 dilution), or anti b-actin (8H10D10) (1:1,000) antibodies were from Cell Signaling Technology (Dancers, USA); anti-DNA polymerase theta antibody (1C11) (1:1,000 dilution) was from Novus Biologicals (Centennial, USA); and anti-NrF2 (ab137550) (1:1,000 dilution) and anti-Bcl-2 (E17) (1:1,000 dilution) antibodies were from Abcam (Cambridge, UK). Immunoreactive proteins were secondary labeled with ECL™ anti-rabbit IgG antibody (1:10,000) (NA934) (Amersham, Little Chalfont, UK) or with anti-mouse IgG-peroxidase antibody (A9044) (1:8,000) (Sigma-Aldrich, Saint Quentin Fallavier, France), visualized by ECL immunodetection (Amersham, Little Chalfont, UK) and quantified by chemoluminescence analysis.

### Statistical Analysis

Results were expressed as mean ± standard error of the mean (SEM). All data were analyzed with GraphPad Prism v8.4.3 software (GraphPad software, San Diego, CA, USA). The Student’s t-test (two-tailed 95% confidence intervals) was used for comparison of two mean values. A normality test (Agostino–Pearson test) was applied to validate the use of an ANOVA with *post-hoc* Bonferroni’s tests when groups of mean values were more than 2. In different figure legends, samples sizes were indicated, and statistical significance was defined as p<0.05.

## Results

### Combined Effect of OxA and Gemcitabine on Cancer Cell Growth

**Figure 1:**
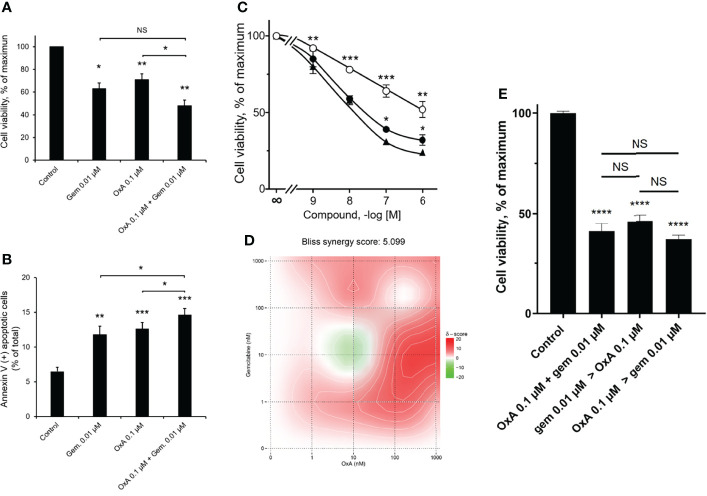
Inhibition of AsPC-1 cell growth by OxA and/or gemcitabine. **(A)** Inhibition of AsPC-1 cell viability induced by 0.01 µM gemcitabine (gem), 0.1 µM OxA (OxA), or 0.01 µM gemcitabine plus 0.1 µM OxA. Results were expressed in the percentage of maximum obtained in the absence of treatment (control). **(B)** Effect of OxA or gemcitabine or their combination on apoptosis of ASPC-1 cells. Apoptosis was determined by annexin V/7-AAD binding, and results were expressed as the percentage of apoptotic cells. **(C)** Effect of the dose–response of OxA or gemcitabine or OxA plus gemcitabine on cell viability of AsPC-1 cells. **(D)** Bliss model diagram and estimation of Bliss synergy score. **(E)** OxA plus gemcitabine treatment and sequential treatment by OxA>gemcitabine or gemcitabine>OxA compared to control (absence of treatment). Results were expressed in the percentage of maximum obtained in the absence of treatment. Data were the means ± SEM of three separate experiments. (○) OxA, (●) gemcitabine, and (▲) OxA plus gemcitabine. NS, non-significant, *p<0.05, **p<0.01, ***p<0.001, and ****p<0.0001 corresponding to comparison with control.

As previously reported, OxA was able to induce a cell apoptosis through the recruitment of the tyrosine phosphatase SHP2 in pancreatic cancer cell line AsPC-1 (19). The pro-apoptotic action of OxA or gemcitabine, which represents a gold standard in the first-line treatment of metastatic PDAC (1), and their combinations were investigated. After 48 h of treatment with OxA or gemcitabine or their combination, AsPC-1 pancreatic cancer cells showed an inhibition of cell viability (**Figure 1A**). Gemcitabine (0.01 µM) or OxA (0.1 µM) reduced the cell growth of about 40%. Moreover, the addition of OxA (0.1 µM) to gemcitabine (0.01 µM) inhibited the cell viability of about 50% (**Figure 1A**). As expected, inhibition of cancer cell growth induced by OxA and gemcitabine resulted in apoptotic action of these compounds (**Figure 1B**). Gemcitabine (0.01 µM) induced a cell apoptosis of 12 ± 1%, 0.1 µM of OxA induced a cell apoptosis of 13 ± 1%, and the combination of these two compounds induced a cell apoptosis of 15 ± 1% (**Figure 1B**). It should be noted that the combination of both treatments was significantly different (p<0.05) from OxA or gemcitabine alone (**Figure 1B**). As shown in **Figure 1C**, the action of OxA or gemcitabine or the combination of OxA and gemcitabine decreased cell growth in a dose-dependent manner. To determine if the action of combined OxA and gemcitabine was additive or synergic, a Bliss synergy score was determined (**Figure 1D**). Currently, surface plot based on Bliss independence response is used to test anticancer synergic action of two independent drugs that targeted two different signaling pathways (23). The resulting Bliss synergy score can be interpreted as the average excess response due to drug interactions (23). In case of the additive action of two drugs, the Bliss synergy score was from −10 to 10 (23). In contrast, a Bliss synergy score >10 indicated that the interaction between these two drugs was synergic. Analysis of dose–response curves encompassing the cell viability action of OxA or gemcitabine and the combination of OxA plus gemcitabine revealed a Bliss synergy score of 5.099, suggesting that OxA and gemcitabine proceeded in an additive way to inhibit the cell growth of pancreatic cell line, AsPC-1 (**Figure 1D**). To investigate the influence of treatment sequence of OxA and gemcitabine on PDAC cell viability, AsPC-1 cells were incubated in the presence of (1) 0.01 µM of gemcitabine for 48 h followed by 0.1 µM of OxA treatment for 48 h (2), 0.1 µM of OxA for 48 h followed by 0.01 µM of gemcitabine treatment for 48 h, or (3) combination treatment for 96 h. As shown in **Figure 1E**, whatever was the treatment sequence, an inhibition of cancer cell viability of about 50% was observed, indicating that there is no influence of treatment sequence on the ability of these two molecules to inhibit cancer cell growth.

### Combined Effect of OxA and NAB-Paclitaxel on Cancer Cell Growth

**Figure 2:**
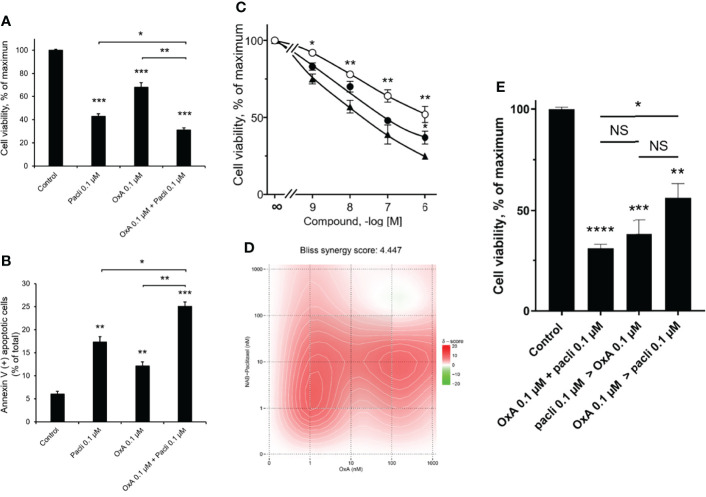
Inhibition of AsPC-1 cell growth by OxA and/or Nab-paclitaxel. **(A)** Inhibition of AsPC-1 cell viability induced by 0.1 µM Nab-paclitaxel (pacli), 0.1 µM OxA (OxA), or 0.1 µM Nab-paclitaxel plus 0.1 µM OxA. Results were expressed in the percentage of maximum obtained in the absence of treatment (control). **(B)** Effect of OxA or Nab-paclitaxel or their addition on apoptosis of ASPC-1 cells. Apoptosis was determined by annexin V/7-AAD binding, and results were expressed as the percentage of apoptotic cells. **(C)** Effect of the dose–response of OxA or Nab-paclitaxel or OxA plus Nab-paclitaxel on cell viability of AsPC-1 cells. **(D)** Bliss model diagram and estimation of Bliss synergy score. **(E)** OxA plus Nab-paclitaxel treatment and Impact of sequential treatment by OxA>Nab-paclitaxel or Nab-paclitaxel>OxA compared to control (absence of treatment). Results were expressed in the percentage of maximum obtained in the absence of treatment. Data were the means ± SEM of three separate experiments. (○) OxA, (●) Nab-paclitaxel, and (▲) OxA plus Nab-paclitaxel. NS, non-significant, *p<0.05, **p<0.01, ***p<0.001, and ****p<0.0001 corresponding to comparison with control.

Currently, Nab-paclitaxel represents a molecule widely used in the treatment arsenal of metastatic PDAC, associated or not to gemcitabine (1). To compare the action of OxA and Nab-paclitaxel on AsPC-1 cell line growth, cells were incubated in the presence of 0.1 µM of OxA or 0.1 µM of Nab-paclitaxel or with a combination of 0.1 µM of each molecule. As shown in **Figure 2A**, the treatment of AsPC-1 by Nab-paclitaxel or OxA or their combinations inhibited cell viability of 57 ± 2%, 32 ± 4%, and 69 ± 2%, respectively. This cancer cell growth inhibition induced by OxA and Nab-paclitaxel resulted in an apoptotic action of these molecules (**Figure 2B**). Nab-paclitaxel (0.1 µM) induced 17.3 ± 1.2% of apoptosis, 0.1 µM of OxA induced 12.1 ± 1.0% of apoptosis, and the combination of these two molecules induced 25.1 ± 1% apoptosis (**Figure 2B**). As expected, the action of OxA or Nab-paclitaxel or their combination on cell viability was dose dependent (**Figure 2C**). The determination of Bliss synergy score of the cell viability dose–response curves of OxA or Nab-paclitaxel or OxA plus Nab-paclitaxel displayed a score of 4.044, indicating that OxA and Nab-paclitaxel were acting in an additive manner (**Figure 2D**). The treatment sequence influence of OxA and Nab-paclitaxel on cancer cell viability was tested as previously described above. As shown in **Figure 2E**, no significant differences were observed between the sequence of Nab-paclitaxel treatment followed by OxA treatment compared to the addition of OxA followed by Nab-paclitaxel treatment. In contrast, a significant difference was obtained between the sequence OxA treatment followed by Nab-paclitaxel treatment compared to addition of OxA and Nab-paclitaxel, indicating that the additive treatment was more favorable than the sequential treatment (**Figure 2E**).

### Impact of OxA and Gemcitabine on Tumor Growth in Preclinical Models

**Figure 3:**
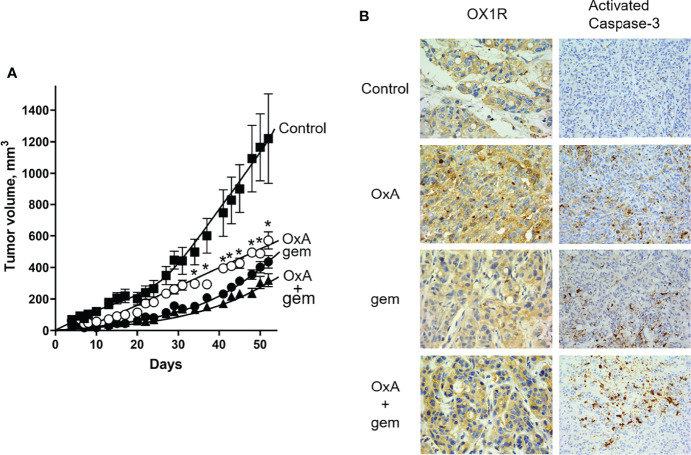
Preclinical study and histological analysis of anti-tumoral effect of OxA and gemcitabine. **(A)** Impact of three injections/week of 100 µg OxA, 120 µg gemcitabine, or 100 µg OxA plus 120 µg gemcitabine on volume of tumors developed from subcutaneously xenografted ASPC-1 cells in nude mice. The tumor development was measured with a caliper. **(B)** Determination of OX1R expression and caspase-3 activation by histological immunostaining in xenografted AsPC-1 resected tumors from mice treated with PBS (control), OxA (OxA), gemcitabine (gem), or OxA plus gemcitabine (OxA + gem). Magnification was 40× (OX1R expression) and 20× (caspase-3 activation). Data were the means ± SEM of six tumors in each group. *p<0.05.

One hundred micrograms of OxA (4 mg/kg) or 120 µg gemcitabine (5 mg/kg) or 100 µg OxA plus 120 µg gemcitabine was intraperitoneally injected three times/week in nude mice subcutaneously xenografted with 2.10^6^ AsPC-1 cells at day 1 after implantation. As shown in **Figure 3A**, OxA and gemcitabine induced a significant and similar inhibition of tumor volume of 56% and 65%, respectively, as compared to untreated control mice. Treatment with the combination of OxA and gemcitabine revealed a significant inhibition of tumor volume of 74% as compared to untreated control mice. Moreover, the combination was significantly more effective than OxA treatment alone (**Figure 3A**). After animal sacrifice and tumor resections, histological analyses by anti-OX1R immunostaining revealed that the OX1R expression was not affected by OxA or gemcitabine or OxA plus gemcitabine treatments as compared to tumors resected from control mice (**Figure 3B**). However, an intense staining of activated cleaved caspase-3 revealing a cell apoptosis was observed in OxA, gemcitabine, and OxA plus gemcitabine treatment, whereas no immunostaining of activated caspase-3 was observed in control mice (**Figure 3B**).

### Impact of OxA and Nab-Paclitaxel on Tumor Growth in Preclinical Models

**Figure 4:**
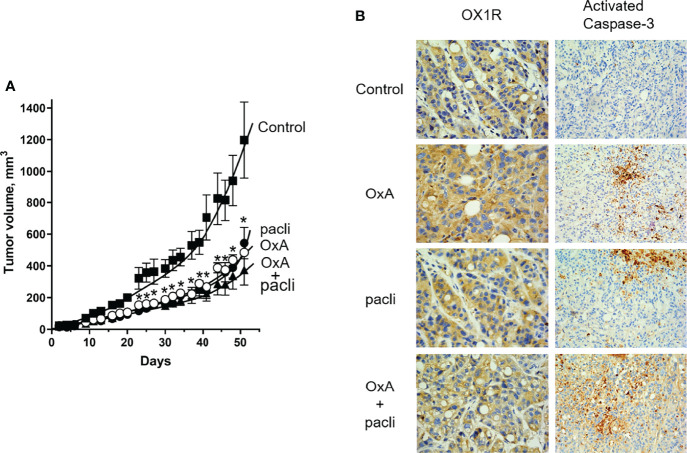
Preclinical study and histological analysis of anti-tumoral effect of OxA and Nab-paclitaxel. **(A)** Impact of three injections/week of 100 µg OxA, 230 µg Nab-paclitaxel, or 100 µg OxA plus 230 µg Nab-paclitaxel on volume of tumors developed from subcutaneously xenografted ASPC-1 cells in nude mice. The tumor development was measured with a caliper. **(B)** Determination of OX1R expression and caspase-3 activation by histologic immunostaining in xenografted AsPC-1 resected tumors from mice treated with PBS (control), OxA (OxA), Nab-paclitaxel (pacli), or OxA plus Nab-paclitaxel (OxA + pacli). Magnification was 40× (OX1R expression) and 20× (caspase-3 activation). Data were the means ± SEM of six tumors in each group. *p<0.05.

After subcutaneous injection of 1.10^6^ AsPC-1 cells in nude mice, intraperitoneal injection three times/week of 100 µg OxA (4 mg/kg) or 230 µg (9.2 mg/kg) Nab-paclitaxel and 100 µg OxA plus 230 µg Nab-paclitaxel on day 1 after implantation showed that OxA and Nab-paclitaxel were able to significantly reduce the tumor volume of 60% and 55%, respectively, as compared to control mice (**Figure 4A**). Moreover, intraperitoneal injection of OxA and Nab-paclitaxel reduced the tumor volume of 70% as compared to control mice. Although statistically insignificant, the addition of OxA to Nab-paclitaxel induced a higher trend of tumor volume reduction than the use of OxA or Nab-paclitaxel alone (**Figure 4A**). Histological analyses of resected tumors revealed that OX1R expression was not modified in Nab-paclitaxel and OxA plus Nab-paclitaxel treatments as compared to control tumors, indicating that different treatments did not have any impact on OX1R expression (**Figure 4B**). Furthermore, injection of OxA or Nab-paclitaxel and OxA plus Nab-paclitaxel induced an intense cell death by mitochondrial apoptosis in treated tumors but not in control tumors (**Figure 4B**).

### Impact of OxA on Tumors Having Been Made Resistant to Gemcitabine or Nab-Paclitaxel

**Figure 5:**
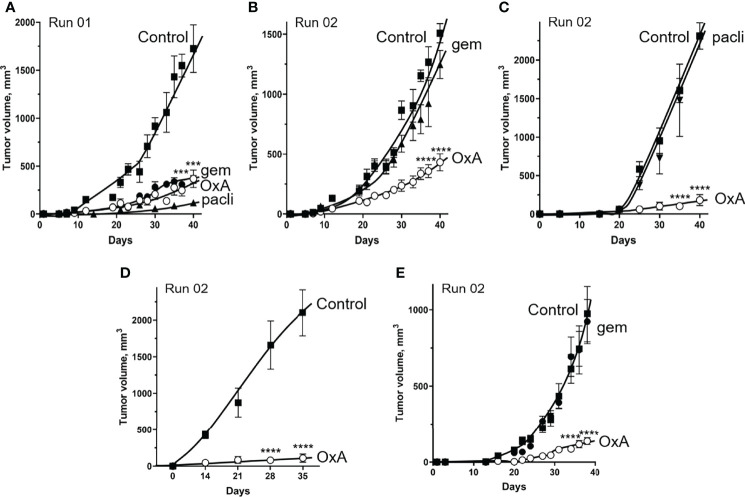
Effect of OxA on tumor development of gemcitabine- and Nab-paclitaxel-chemoresistant tumors xenografted in nude mice. **(A)** A total of 2.10^6^ AsPC-1 cells were subcutaneously injected in nude mice, which were treated by three injections/weeks of PBS (control), OxA (OxA), gemcitabine (gem), or Nab-paclitaxel (pacli). This first step of the experiment was noted as “Run 01.” After 40 days, mice were sacrificed, and resected tumors were dissociated with collagenase and subcutaneously reinjected in nude mice. This step was named “Run 02” of experiments. **(B)** The impact of OxA and gemcitabine on tumor development of gemcitabine-treated tumors xenografted in nude mice (Run 02). **(C)** Impact of OxA and Nab-paclitaxel on tumor development of NAB-paclitaxel-treated tumors xenografted in nude mice (Run 02). **(D)** Impact of OxA on tumor development of OxA-treated tumors xenografted in nude mice (Run 02). **(E)** The impact of OxA and gemcitabine (Run 02) on tumor development of gemcitabine-treated tumors from Run 01 experiment in which tumors were obtained by xenografting of 2.10^6^ PDAC15 (PDX) cells. Data were the means ± SEM of six tumors in each group. ***p<0.001 and ****p<0.0001.

Tumors from mice xenografted with AsPC-1 cells, having been treated by gemcitabine or Nab-paclitaxel or OxA (run01) for 40 days (**Figure 5A**), were resected, dissociated with collagenase IV, and subcutaneously reinjected in nude mice to form new tumors (run02). As shown in **Figure 5B**, injection of gemcitabine did not inhibit run02 tumor growth as compared to control, suggesting that run02 tumors had acquired gemcitabine resistance. In contrast, OxA was always able to inhibit the tumor’s growth obtained with the cell of run01 (**Figure 5B**), suggesting that OxA reduced the volume of gemcitabine-resistant tumors. Similarly, injection of Nab-paclitaxel had no effect on tumor growth of run02 as compared to control (**Figure 5C**), indicating that these tumors displayed resistance to Nab-paclitaxel. However, injection of OxA induced a reduction in tumor volume in Nab-paclitaxel-resistant tumors (**Figure 5C**). Moreover, no resistance to OxA treatment was observed in tumors. As shown in **Figure 5D**, tumors developed (run02) from previous OxA-treated tumors (run01) were fully sensitive to OxA. It should be noted that the OxA sensitivity of gemcitabine-resistant tumors was also observed for tumors developed from patient-derived xenograft (PDX) cells isolated from PDAC tumors. Indeed, cells from this PDX (PDAC15), which has been previously characterized as OX1R-expressing cells (22), were subcutaneously injected in nude mice followed by gemcitabine treatment (run01), and cells from resulting tumors were again injected to nude mice. As shown in **Figure 5E**, OxA was also able to reduce tumors volume of gemcitabine-resistant tumors from PDX.

**Figure 6:**
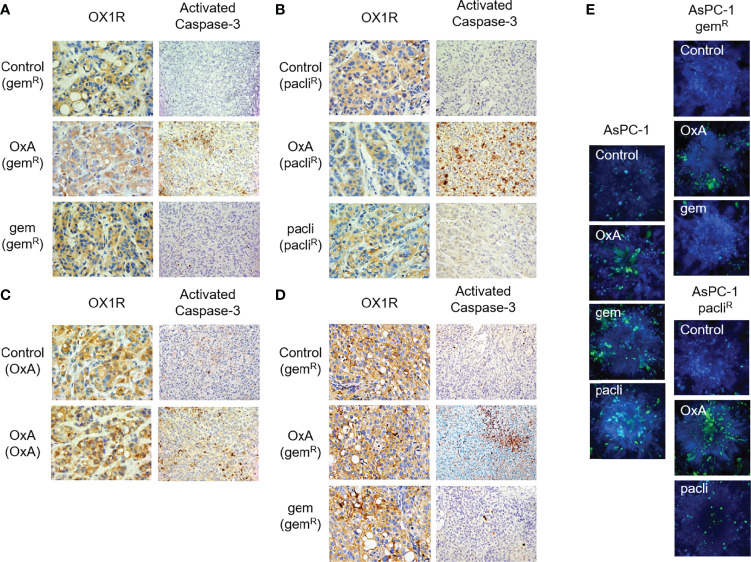
Histological analysis of resected tumors from Run 02 experiments. **(A)** OX1R expression and caspase-3 activation in gemcitabine-chemoresistant AsPC-1 tumors (gem^R^) treated with PBS (Control), OxA (OxA), and gemcitabine (gem). **(B)** OX1R expression and caspase-3 activation in Nab-paclitaxel-chemoresistant AsPC-1 tumors (pacli^R^) treated with PBS (Control), OxA (OxA), and Nab-paclitaxel (pacli). **(C)** OX1R expression and caspase-3 activation in 50 days OxA-treated tumors (OxA) treated with PBS (control) and OxA (OxA). **(D)** OX1R expression and caspase-3 activation in gemcitabine-chemoresistant tumors obtained by xenografting of 50 days gemcitabine-treated PDAC15 cells (gem^R^) treated with PBS (control), OxA (OxA), and gemcitabine (gem). **(E)** Cell viability determination of spheroids developed with parental AsPC-1, gemcitabine-resistant, and Nab-paclitaxel-resistant AsPC-1 cells isolated in Run 02 experiment. Magnification was 40× (OX1R expression) and 20× (caspase-3 activation).

Histological analyses show that OX1R was expressed in gemcitabine and Nab-paclitaxel tumors (**Figures 6A, B**). This expression was not modified by OxA and gemcitabine or Nab-paclitaxel treatment (**Figures 6A, B**). However, only OxA was able to induce the caspase-3 activation in tumor tissues as compared to control and gemcitabine or Nab-paclitaxel-treated tumors (**Figures 6A, B**). In addition, OX1R was expressed in tumors obtained with cells from tumors previously treated by OxA (**Figure 6C**). Moreover, the activation of caspase-3 was observed when the tumors were treated with OxA as compared to control tumors (**Figure 6C**). In the same way, OX1R was expressed in gemcitabine-resistant tumors from PDAC15, and OxA was able to activate caspase-3 in these tumors as compared to control tumors (**Figure 6D**). This set of results indicates that OxA was able to inhibit tumor growth of gemcitabine- or Nab-paclitaxel-resistant tumors involving a proapoptotic process, in preclinical models using PDAC cell lines or PDX.

To confirm these observations, spheroids were developed with AsPC-1 cells isolated from control, gemcitabine-resistant, or Nab-paclitaxel-resistant tumors (run02); then, they were treated with PBS, OxA, gemcitabine, or Nab-paclitaxel (**Figure 6E**). After 7 days of culture, the cell viability of spheroids was tested using ReadyProbes Cell Viability Imaging Kit in which live cells were associated to blue fluorescence and dead cells were associated with green fluorescence. As shown in **Figure 6E**, spheroids, in the absence of treatment (control), were mostly composed of living cells, whereas in the presence of OxA, gemcitabine, and Nab-paclitaxel, a lot of dead cells appeared. In contrast, spheroids obtained from gemcitabine-resistant tumor cells displayed a lack of dead cells when spheroids were untreated or treated with gemcitabine (**Figure 6E**), while dead cells appeared in the presence of OxA (**Figure 6E**). Under the same conditions, spheroids from Nab-paclitaxel-resistant tumor cells were insensitive to Nab-paclitaxel treatment as compared to untreated spheroids (**Figure 6E**). In contrast, OxA induced cell death in these spheroids (**Figure 6E**).

### OxA Induced an Increase in ATF3 and Cleaved PARP in Gemcitabine- and Nab-Paclitaxel-Resistant Tumors

**Figure 7:**
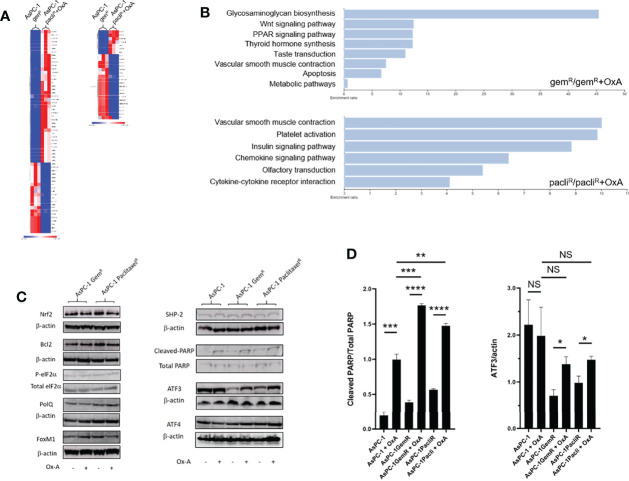
RNA-seq and Western blot analysis of gemcitabine- and Nab-paclitaxel-chemoresistant tumors treated or not by OxA. **(A)** Heatmap of mRNA expression in gemcitabine- and Nab-paclitaxel-chemoresistant tumors treated or not by OxA. **(B)** Diagram of functional enrichment analysis of mRNA expression in gemcitabine- and Nab-paclitaxel-chemoresistant tumors treated or not by OxA. **(C)** NrF2, Bcl2, p-elF2α, PolQ, FoxM1, SHP-2, cleaved-PARP, ATF3, and ATF4 factor expression in gemcitabine- and Nab-paclitaxel-chemoresistant tumors treated or not by OxA. **(D)** Semi-quantitative analysis of cleaved-PARP and ATF3 expression in gemcitabine- and Nab-paclitaxel-chemoresistant tumors treated or not by OxA, determined by Western blot.

To determine whether OxA induced tumor growth inhibition *via* OX1R expression in gemcitabine- or Nab-paclitaxel-resistant tumors or/and modified the expression of proteins involved in the resistance processes, a transcriptomic analysis of expression of various proteins related to drug resistance in PDAC was carried out. Transcripts specifically regulated in gemcitabine- or Nab-paclitaxel-resistant tumors treated or not by OxA were identified by RNA-seq and analyzed by functional enrichment using GSEA software and WEB-based GEne SeT AnaLysis Toolkit (WebGeSTAT) (22). As shown in **Figure 7A**, few transcripts were impacted by OxA in gemcitabine- or Nab-paclitaxel-resistant tumors. These transcripts mainly represent pseudogene and long non-coding RNA (lncRNA). Nevertheless, OxA was able to increase the expression of HRK coding for harakiri protein, which promoted apoptosis by interaction with Bcl-2 and Bcl-XL (24). Moreover, functional enrichment analysis using WikiPathway cancer database showed that differential expression of transcripts associated with OxA treatment in AsPC-1 cells resistant to gemcitabine or Nab-paclitaxel were related to apoptosis, Wnt signaling in cancer, olfactory transduction, and insulin or chemokine signaling pathways (**Figure 7B**). These results indicated that OxA treatment of gemcitabine- or Nab-paclitaxel-resistant tumors had very small impact on transcriptome excepted for HRK, which displayed pro-apoptotic properties. The expression of various proteins expressed in PDAC and participating in tumoral drug resistance including cell stress proteins (Nrf2, p-eIF2α, ATF3, and ATF4), DNA reparation proteins (PolQ and PARP), oncogenic protein (FoxM1), and anti-apoptotic protein (Bcl2) were analyzed in gemcitabine- or Nab-paclitaxel-resistant tumors treated or not by OxA. As shown in **Figure 7C**, the expression of Nrf2, Bcl2, peIF2α, PolQ, FoxM1, and ATF4 was not modified in the presence of OxA in gemcitabine- or Nab-paclitaxel-resistant tumors. It should be noted that the expression of SHP2, which played a key role in pro-apoptotic action of orexins in digestive cancers (15, 19), was not regulated by OxA (**Figure 7C**). In contrast, OxA was able to increase the expression of ATF3 in gemcitabine- or Nab-paclitaxel-resistant tumors. However, in non-resistant AsPC-1 cells, ATF3 was highly expressed, whereas in gemcitabine- or Nab-paclitaxel-resistant cells, ATF3 was much lower expressed (**Figure 7C**). Semi-quantitative analysis of Western blot showed that OxA also induced the expression of ATF3 in gemcitabine- or Nab-paclitaxel-resistant cells (**Figure 7D**, right panel). As PARP was involved in DNA reparation and cell survival of cancer cells (25), we determined the action of OxA on PARP cleavage leading to its inactivation, in gemcitabine- or Nab-paclitaxel-resistant tumors. As shown in **Figure 7C**, OxA induced an increase of PARP cleavage in gemcitabine- or Nab-paclitaxel-resistant tumors. Semi-quantitative analysis of Western blot confirmed these observations and revealed that OxA increased the cleavage of PARP in gemcitabine- or Nab-paclitaxel-resistant but not in non-resistant AsPC-1 cells (**Figure 7D**, left panel).

## Discussion

It is well known that GPCRs, identified as the largest cell surface receptor family that is the most common therapeutic target encompassing about 40% of FDA-approved molecules, represent an innovative target in cancer (26). It is common that GPCRs emerged as key targets in tumor growth and/or metastasis associated to their overexpression/underexpression in cancer cells and also their abilities to stimulate/inhibit proliferation or inversely stimulate/inhibit apoptosis (27). Among this large family, we have demonstrated in 2011 that OX1R, but not OX2R, was ectopically expressed in colon cancer whatever its location and/or grade of development (15). The OX1R activation in colon cancer cells by exogenous orexins (OxA or OxB) induced the mitochondrial caspase-dependent apoptosis (28) The OxA/OX1R system was responsible for the anti-tumoral impact in preclinical models in which colon cancer cells were xenografted and also in preclinical models where pancreatic or liver cancer cells were xenografted (15, 19, 29). Usually, the first or second line of pancreatic cancer treatment, notably in metastatic pancreatic cancer, involved gemcitabine and Nab-paclitaxel (1). Here, the comparison between gemcitabine, Nab-paclitaxel, and OxA treatment on the OX1R-expressing pancreatic cancer cell line, AsPC-1, showed that OxA had a similar impact in terms of cancer cell growth as compared to gemcitabine and Nab-paclitaxel. Moreover, the addition of OxA to gemcitabine or Nab-paclitaxel improved the impact of those molecules. These potentiation effects were associated to an additive effect and not to a synergistic effect. The absence of synergic effect observed between OxA plus gemcitabine or OxA plus Nab-paclitaxel could be related to the different mechanisms of action of these three molecules. In fact, OxA/OX1R directly activates mitochondrial apoptosis in pancreatic cancer cells, whereas gemcitabine inhibited the DNA synthesis in pancreatic cancer cells, inducing cytotoxicity that leads to apoptosis (30), and Nab-paclitaxel inhibited microtubule dynamics inducing cytotoxicity that also leads to apoptosis (12). It should be noted that the sequences of first treatment by OxA followed by gemcitabine or inversely were not significantly different than the simultaneous addition of OxA and gemcitabine. In contrast, the sequence of first treatment by OxA followed by Nab-paclitaxel was significantly less effective than the simultaneous addition of OxA and Nab-paclitaxel, suggesting that the pro-apoptotic action of OxA in the first was not favorable to Nab-paclitaxel’s following action. These observations reinforced the fact that the inhibition of AsPC-1 cell growth by OxA plus gemcitabine or OxA plus Nab-paclitaxel was fully additive and also indicated that the addition of OxA with gemcitabine or with Nab-paclitaxel was more effective than molecules alone. Our previous data demonstrated that OxA reduced tumor volume of subcutaneously xenografted mice with AsPC-1 cells (19). In the present report, we confirmed that OxA strongly reduced the tumor volume of xenografted mice with AsPC-1 cells and show that this anti-tumoral effect was similar to gemcitabine or Nab-paclitaxel treatment. Although statistically not significant, it seems that the treatment by OxA plus gemcitabine or OxA plus Nab-paclitaxel was more effective than OxA alone, indicating that OxA, *in vivo*, also had an additive effect with the two drugs. It was well known that repetitive activation of GPCRs by agonist ligands led to a decrease in GPCR response associated to desensitization and cell internalization processes (31). However, histological analysis of xenografted tumors treated over the long term, with OxA, gemcitabine, Nab-paclitaxel, OxA plus gemcitabine, or OxA plus Nab-paclitaxel revealed that OX1R was always expressed in tumor cells after 50 days of these different treatments. These observations indicate that OX1R was not downregulated and/or the receptor turnover maintained OX1R expression at the cell surface in the presence of these three molecules. As expected, OxA-treated xenografted tumors revealed the presence of activated caspase-3, demonstrating that OxA-induced tumor volume reduction was associated to mitochondrial apoptosis (15, 19). Moreover, xenografted tumors treated with gemcitabine or Nab-paclitaxel displayed activated caspase-3, showing that these two drugs induced caspase-dependent apoptosis as previously reported in various models (12, 30).

The activation of OX1R by exogenous OxA induced a strong reduction in tumor volume in mice xenografted with AsPC-1 cells displaying a chemoresistance to gemcitabine or Nab-paclitaxel. In contrast, no resistance to OxA treatment has been identified to be probably associated to a permanent OX1R expression in xenografted tumors and the ability of this receptor to induce apoptosis after its activation by OxA. It should be noted that OX1R was also expressed in colon cancer cell line, HT29-FU, which presented resistance to 5-FU, established *in vitro*, and the activation of OX1R by OxA induced a pro-apoptotic effect in these cells (15). Histological analysis of gemcitabine- and Nab-paclitaxel-resistant xenografted tumors treated by OxA revealed a caspase-3 activation, demonstrating the induction of apoptosis by OxA in chemoresistant tumors. We have demonstrated that OX1R was expressed in PDX named PDAC15 isolated from a patient’s tumor and that the activation of OX1R in PDAC15 led to a tumor growth inhibition in preclinical models (19). Here, we observed that OxA induced a tumor development inhibition in gemcitabine-resistant tumors xenografted with PDAC cells isolated from tumor patient (PDX). Taking these observations into account, we demonstrated that OX1R was already expressed in chemoresistant tumors and that its activation induced an anti-tumoral effect.

RNA sequence analysis of mRNA prepared from gemcitabine-resistant or Nab-paclitaxel-resistant PDAC cells isolated from tumors that developed in xenografted mice, treated or not by OxA, revealed that OxA had a poor effect on the transcriptome of these cells. The major impact was mainly focused on pseudogenes and lncRNA. This observation was confirmed by the functional enrichment analysis, which showed that the major impact was related to the markers of signaling pathways, such as Wnt, insulin, chemokine, and olfactory signaling pathways. However, in gemcitabine-resistant tumors treated with OxA, the apoptosis signaling pathway represented by an increase in HKR transcripts was revealed. HRK, also named harakiri, expressed in the pancreas, liver, lung, kidney, and prostate, was an activator of apoptosis through the inhibitory interaction with Bcl-2 and Bcl-X, which had anti-apoptosis properties (24). HRK gene seems to have an important role in the apoptosis regulation in tumor cells, and its inactivation by methylation could be related to tumorigenesis of prostate cancer (32). However, it cannot exclude that the overexpression/underexpression of lncRNA and/or pseudogenes in OxA-treated gemcitabine-resistant and Nab-paclitaxel-resistant PDAC cells could play a role in anti-tumoral action of OxA. Indeed, it was demonstrated that particular lncRNA or pseudogenes played a role in cancer by interacting with chromatin, proteins, and RNAs in cancer cells (33). The impact of OxA treatment on chemoresistant tumors, on the expression of various proteins such as Nrf2, p-eIF2α, ATF4, PolQ, FoxM1, and Bcl2, which are involved in cancer chemoresistance (34–38), is not shown. Interestingly, neither OX1R expression nor SHP2, which displayed a key role in cell apoptosis induced by orexins (14), was regulated by OxA. In contrast, OxA was able to increase the expression of ATF3 in gemcitabine- and Nab-paclitaxel-resistant tumors. Cyclic AMP-dependent transcription factor ATF-3 is also playing a role in modulating metabolism and stress in various tissues including the pancreas, liver, heart, hypothalamus, and adipose tissue (39). In stress condition, ATF3 was able to regulate the expression of Noxa and Bnip3, which are involved in pro-apoptotic process. In the colon, liver, and prostate cancers, ATF3 had tumor suppression impact by apoptosis activation (39). In PDAC, ATF3 increased the chemosensitivity of gemcitabine treatment (40). The overexpression of ATF3 induced by OxA on chemoresistant tumors could reinforce the anti-tumoral impact of orexins by increasing apoptosis. Moreover, OxA was able to increase the cleavage of PARP [poly (ADP-ribose) polymerase], which is a protein family involved in DNA repair and genomic stability, and was inactivated by caspase cleavage during the apoptotic pathway (41, 42). The PARP cleavage observed in gemcitabine- and Nab-paclitaxel-resistant tumors treated by OxA was likely a consequence of orexins caspase activation.

In conclusion, the activation by orexins of OX1R, which was expressed in non-resistant and resistant PDAC to gemcitabine or Nab-paclitaxel, induced important pro-apoptotic and anti-tumoral actions. It is of note that one current innovative therapeutical strategy is to reactivate mitochondrial apoptosis by action of new compounds as taxanes, BH3 mimetic, and metformin (32). In that respect, OxA or molecules derived from orexins represent emerging innovative therapeutical compounds with great potential interest in the treatment of PDAC and chemoresistant pancreatic cancers.

## Data Availability Statement

The raw data supporting the conclusions of this article will be made available by the authors, without undue reservation.

## Ethics Statement

The studies involving human participants were reviewed and approved by the Institutional Review Board (CEERB GHU Paris Nord Nos. IRB12-059 and 12-033). The patients/participants provided their written informed consent to participate in this study. The animal study was reviewed and approved by Apafis No. 17199-201810221522166v4.

## Author Contributions

ACn, TV, and PN designed the experiments. ACn, TV, VG, and PN performed experiments. ACd, ACn, ACh, VR, TV, and DM have performed and analyzed histologic studies. ACn has written the manuscript. All authors have read, edited and approved the final version of manuscript.

## Funding

Our work was supported by the “Institut National de la Santé et de la Recherche Medicale” (INSERM), the “Université de Paris,” The “Institut National du Cancer (INCA)” (PAIR Pancreas, grant number PAN18-045), and the “Ligue Contre le Cancer” (grant numbers R16020HH and GB/MA/CD/EP-12062).

## Conflict of Interest

The authors declare that the research was conducted in the absence of any commercial or financial relationships that could be construed as a potential conflict of interest.

## Publisher’s Note

All claims expressed in this article are solely those of the authors and do not necessarily represent those of their affiliated organizations, or those of the publisher, the editors and the reviewers. Any product that may be evaluated in this article, or claim that may be made by its manufacturer, is not guaranteed or endorsed by the publisher.
